# Ginsenoside Rg1 Drives Stimulations of Timosaponin AIII-Induced Anticancer Effects in Human Osteosarcoma Cells

**DOI:** 10.1155/2020/8980124

**Published:** 2020-07-22

**Authors:** Sang Yeol Lee

**Affiliations:** Department of Life Science, Gachon University, San 65, Bokjeong-Dong, Sujeong-Gu, Seongnam-Si, Gyeonggi-Do 461-701, Republic of Korea

## Abstract

A ginsenoside Rg1 is an active compound extracted from the stem and/or root of ginseng. Rg1 has been known to affect various human organ systems including the immune, cardiovascular, and nervous systems with its pharmacological effects. Timosaponin AIII (TA3) is a type of spirostanol saponins that are the major compounds of *Anemarrhena asphodeloides*. TA3 exerts anticancer effects in various human cancers, and the effects include attenuations of cancer cell migration and induction of apoptosis. In this study, I report that Rg1 drives the stimulation of TA3-induced cytotoxic effects in MG63 human osteosarcoma cells. Rg1 stimulates TA3-induced apoptosis in MG63 cells via selective intensification of caspase-3 activation. Rg1 and TA3 synergistically induced antimetastatic effects such as attenuation of MG63 cell migration and inhibitions of matrix metalloproteinases (MMP-2 and MMP-9). Rg1 and TA3 synergistically suppressed JNK, p38, ERK, *β*-catenin, and CREB signaling, which are key regulators of cancer metastasis. Finally, the synergistic anticancer effects of Rg1 and TA3 were also observed in U2OS human osteosarcoma cells, and this may indicate that the synergy is not limited specifically to MG63 cells. The results presented here suggest that the combinatorial use of Rg1 and TA3 may be a promising way to develop an effective antiosteosarcoma agent.

## 1. Introduction

**Figure 1:**
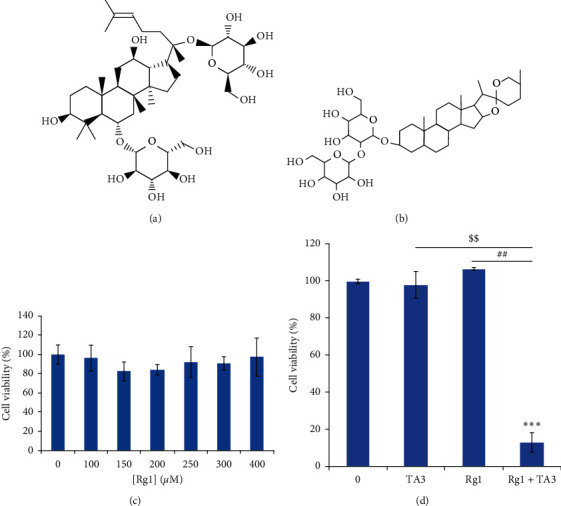
Synergistic cytotoxic effect of Rg1 and TA3 on MG63 human osteosarcoma cells. (a) Rg1 chemical structure. (b) Chemical structure of Timosaponin AIII. (c) The viability of Rg1-treated MG63 cells was investigated by performing the viability assay using the CCK-8 kit. The cells were incubated with Rg1 (0, 100, 150, 200, 250, 300, and 400 *μ*M) for 24 h. (d) Cytotoxic effect of Rg1 plus TA3 was monitored in MG63 cells. The cells were treated with each chemical compound for 24 h: TA3 (6 *μ*M), Rg1 (250 *μ*M), TA3 (6 *μ*M) plus Rg1 (250 *μ*M) for 24 h. Then the samples were subjected to the cell viability assay using CCK-8 kit. The results were statistically evaluated using ANOVA and Tukey's test (^*∗∗∗*^*P* < 0.001 vs control, ^$$^*P* < 0.01 vs treatment of TA3, ^##^*P* < 0.01 vs treatment of Rg1).

Ginseng has been widely used as an effective herbal medicine in Northeast Asian countries. Among the various active components, ginsenosides are the representative phytochemicals that are attributable to the pharmacological effects of ginseng, particularly anticancer activities [1]. Of various ginsenosides known so far, Rg1 is extracted from the ginseng root or stem (Figure 1(a)) [2, 3]. Rg1 shows pharmacological effects in various organ systems such as the nervous and immune systems [4, 5]. It is particularly known to reduce apoptosis of nerve cells and mesenchymal stem cells [6, 7].

Among the various types of human cancers, osteosarcoma develops in the bone. It is one of the most aggressive cancers. The 5-year survival rate of osteosarcoma is near 60% [8]. Despite the efforts to develop effective antiosteosarcoma drugs, the survival rate has been continuously decreased so far [9]. This raises the necessity to develop a highly effective way of cure for human osteosarcoma.

Timosaponin AIII (TA3) is separated from *Anemarrhena asphodeloides* and is classified as a spirostanol saponin. At the C3 position of TA3, a sugar chain is attached (Figure 1(b)) [10]. It was reported that TA3 attenuates invasion and migration of A549 human non-small-cell lung cancer cells via the regulation of matrix metalloproteinases (MMP-2 and MMP-9) [11]. It also triggers autophagy in HeLa cells and regulates the proliferation of human prostate and breast cancer cells [12, 13]. Recently, it was reported that TA3 has antimetastatic and apoptosis-inducing effects in MG63 human osteosarcoma cells [14].

In developing a new anticancer compound, multidrug resistance (MDR) has been one of the greatest hurdles. Therefore, there have been efforts to overcome MDR [15]. As a part of these efforts, various active compounds from ginseng were selected and tested to improve other effective compounds to solve MDR [16, 17].

In this study, I aimed to investigate the anticancer effects of Rg1 on MG63 human osteosarcoma cells and its possible synergy with TA3. Rg1 exerts stimulatory effects on TA3-induced cytotoxic effect and apoptosis in MG63 cells. Rg1 also escalates antimetastatic effects induced by TA3. The combination of Rg1 and TA3 may be a strong candidate for an effective antiosteosarcoma agent.

## 2. Materials and Methods

### 2.1. Cell Culture

MG63 and U2OS human osteosarcoma cells were cultured in Dulbecco's modified Eagles' medium (DMEM) (HyClone, South Logan, UT, USA). DMEM was supplemented with 10% fetal bovine serum (FBS) (Sigma, St. Louis, MO, USA) and antibiotics (100 U/mL of penicillin and 100 mg/mL streptomycin) (HyClone). Both cells were incubated at 37°C in a humidified atmosphere of 5% CO_2_. TA3 (Santa Cruz, CA, USA) was dissolved in dimethyl sulfoxide (DMSO) (Sigma) to prepare 10 mM stock solutions.

### 2.2. Cell Viability Assay

To investigate the cytotoxic effect of Rg1 (Ace EMzyme, Anseong, Korea) on human osteosarcoma cells, Cell Counting Kit-8 (CCK-8) assay was performed as per the manufacturer's protocol (Dojindo Molecular Technologies, Inc., Rockville, MD, USA). MG63 and U2OS cells were seeded in 96-well plates at densities of 1 × 10^4^ cells per well along with DMEM supplemented with 10% FBS. Cells were incubated overnight. Then, MG63 cells were treated with various dosages of Rg1 (0, 100, 150, 200, 250, 300, and 400 *μ*M) for 24 h. To observe the effect of Rg1 on TA3-induced cytotoxicity, MG63 and U2OS cells were exposed to 6 *μ*M of TA3 (Santa Cruz, CA, USA) and/or 250 *μ*M of Rg1 for 24 h. The cell viability was monitored using a spectrophotometer (Biochrom Ltd., Cambridge, UK) with absorbance at wavelength 450 nm.

### 2.3. Annexin V/PI Staining Apoptosis Assay

The effect of Rg1 on TA3-induced apoptosis was investigated by the Annexin V/PI staining apoptosis assay using the FITC Annexin V apoptosis kit, which was purchased from BD Biosciences (Franklin Lakes, NJ, USA). MG63 and U2OS cells were grown in a 6-well plate overnight (1.5 × 10^5^ cells/well). Then, the cells were treated with 6 *μ*M of TA3 and/or 250 *μ*M of Rg1 for 24 h. The cells, which were treated with TA3 and Rg1, were washed with Dulbecco's phosphate-buffered saline (Sigma). The washed cells were resuspended in 1X binding buffer with FITC Annexin V and PI reagent at 25°C in dark room. After 15 min, the treated cells were analyzed using flow cytometry (Beckman Coulter, USA).

### 2.4. TUNEL DNA Fragmentation Analysis

The fluorescence of apoptotic cells was monitored by the fluorometric TUNEL detection system (Promega, Madison, WI, USA). MG-63 and U2OS cells were seeded in 6-well plates at a density of 2.0 × 10^5^ cells per well and incubated for 24 h. Then, the cells were treated with 6 *μ*M of TA3 and/or 250 *μ*M of Rg1 for 24 h. Then, the cells were fixed with 4% formaldehyde for 25 min at room temperature and permeabilized using 0.2% Triton X-100 for 5 min at room temperature. Following that, the cells were treated with 50 *μ*L TdT reaction mixed solution and incubated at 37°C for 1-2 h. The cell nucleus was stained using a Hoechst stain solution (Invitrogen). One microliter of Hoechst stain solution was dissolved in 2 mL of PBS. Labeled strand breaks were observed by fluorescence microscopy (CKX53; Olympus, Shinjuku, Tokyo, Japan). CXP software (Beckman Coulter, CA, USA) was used to analyze the results. For Annexin V (apoptosis), PI (apoptosis), and DCF-DA (ROS), the absorbance at wavelength 520 nm, 620 nm, and 520 nm was assessed, respectively.

### 2.5. DCF-DA Reactive Oxygen Species (ROS) Detection Assay

To investigate whether Rg1 and/or TA3 affect the reactive oxygen species level in MG63 cells, 2′,7′-dichlorofluorescein diacetate reactive oxygen species assay was conducted. MG63 cells were harvested by trypsinization. Then, the cells were resuspended in 10 *μ*M of 2′,7′-dichlorodihydrofluorescein diacetate (molecular probe) (Invitrogen, Carlsbad, CA, USA) in HBSS. After 10 min at 37°C, the cells were analyzed using flow cytometry (Beckman Coulter, USA).

### 2.6. Wound Healing Migration Assay

MG63 and U2OS human osteosarcoma cells were seeded in a 96-well plate (1.0 × 10^4^ cells/well) and maintained overnight. Then, the middle of the cell surface was scraped with a sterile 200 *μ*l micropipette tip to make a scratch of constant width. The debris was washed with PBS and cells were exposed to 6 *μ*M of TA3 and/or 250 *μ*M of Rg1 in DMEM containing 10% FBS. 0.1% FBS was used for negative control (−). After 24 h, wound closure was monitored and photographed by a Leica DM IL LED (Leica Microsystems, Wetzlar, Germany).

### 2.7. Gelatin Zymography

MG63 and U2OS cells were seeded in a 60 mm cell culture dish (2.0 × 10^5^ cells/well) and maintained overnight. Next day, cells were treated with 6 *μ*M of TA3 and/or 250 *μ*M of Rg1 in the presence of DMEM with 0.1% FBS and incubated for 48 h. Culture media were harvested and proteins from samples were separated by electrophoresis using 8% SDS-polyacrylamide gel with 0.1% gelatin. The gels were rinsed with renaturing buffer (2.5% Triton X-100 in distilled H_2_O) three times for 30 min at room temperature (RT) and incubated in zymography developing solution (50 mM Tris-HCl, pH 7.5, 5 mM CaCl_2_) for 24 h at 37°C. The gels were stained with Coomassie Brilliant blue R Staining Solution for 30 min and washed with destaining solution (20% methanol, 10% acetic acid, and 70% distilled H_2_O) to observe degraded areas.

### 2.8. Quantitative Real-Time Polymerase Chain Reaction

Quantitative real-time polymerase chain reaction (qRT-PCR) was conducted to analyze relative levels of mRNAs. MG63 and U2OS cells were treated with 6 *μ*M of TA3 and/or 250 *μ*M of Rg1 in DMEM containing 0.1% FBS. To extract RNA from cells, HiGene Total RNA Prep kit (Biofact, Korea) was used following the manufacturer's protocol. cDNA was synthesized using HiSenScript RH(−) RT PreMix Kit (Intronbio, Seongnam, Korea). For qRT-PCR, QuantiSpeed Sybr Kit (Philekorea) and ROTER GENE Q (QIAGEN) were used for the determination of cycle threshold (Ct) values. 2^ΔΔ*Ct*^ method was used to calculate the relative gene expressions. Primer sequences used for qRT-PCR are provided in Table 1.

### 2.9. Western Blotting

MG63 and U2OS cells were seeded in 6-well dishes (1.5 × 10^5^ cells/well). Cells were treated with 6 *μ*M of TA3 and/or 250 *μ*M of Rg1 for 24 h. Cells were harvested and lysed in RIPA buffer mixed with phosphatase inhibitor cocktail (Gendepot, USA) and protease inhibitor cocktail (Gendepot). Protein concentrations of cell lysates were normalized by the BCA protein assay kit (Thermo Fisher, USA). SDS-polyacrylamide gel electrophoresis was performed to separate proteins from samples. The proteins on the gel were transferred to polyvinylidene fluoride membrane (Millipore, Billerica, MA). The membranes were incubated in blocking buffer (5% skim milk in tris-buffered saline (TBS) with Tween 20) for 1 h at RT with gentle agitation. Blocking buffer was removed and the membranes were incubated with primary antibodies against various target proteins at 4°C overnight. Primary antibodies against phospho-ERK (sc-7383), ERK (sc-94), JNK (sc-571), phospho-JNK (sc-6254), phospho-p38 (sc-17852-R), p38 (sc-7972), phospho-CREB (sc-7978-R), actin (sc-1615), and *β*-catenin (sc-7199) were purchased from Santa Cruz Biotechnology (Santa Cruz, CA, USA). Primary antibodies against CREB (9104), caspase-3 (9662), and caspase-7 (12827) were obtained from Cell Signaling Technology (Beverly, MA, USA). Next day, the membranes were incubated with secondary antibodies at 4°C for 2 h. Secondary antibodies conjugated with horseradish peroxidase (HRP) were purchased from Bethyl Laboratories, Inc. (Montgomery, TX, USA). The membranes were washed with TBS-T three times. The immunoreaction was observed using the enhanced chemiluminescence (ECL) method. In the ECL method, equal volumes of ECL solution A (2.5 mM Luminol, 0.4 mM *p*-Coumaric acid, 100 mM Tris-HCl, pH 8.5) and ECL solution B (0.02% H_2_O_2_, 100 mM Tris-HCl, pH 8.5) were mixed together. The mixture was added to membranes. X-ray film was exposed to membranes to detect luminescence.

### 2.10. Statistical Analysis

All quantitative data are marked as the mean ± SD from three independent experiments. The statistical significance of the result was analyzed by ANOVA and Tukey's test.

## 3. Results

### 3.1. Synergistic Cytotoxic Effect of Rg1 and TA3 in MG63 Human Osteosarcoma Cells

To investigate the cytotoxic effect of Rg1 on MG63 human osteosarcoma cells, a Cell Counting Kit-8 (CCK-8) assay was performed. The cells were treated with ascending dosages of Rg1 (0, 100, 150, 200, 250, 300, and 400 *μ*M). Then, the cell viability was monitored by CCK-8 assay. As presented in Figure 1(c), the cytotoxic effect was not significant. This result indicates that Rg1 alone is not toxic to MG63 cells. To study the potential cytotoxic effect of the combinatorial application of Rg1 and TA3, CCK-8 assay was carried out after treatment with Rg1 and/or TA3 for 24 h (Figure 1(d)). As seen in Figure 1(d), the combination of Rg1 and TA3 (250 *μ*M and 6 *μ*M, respectively) exerted a significant cytotoxic effect (approximately 13% cells survived), whereas the single applications of Rg1 and TA3 did not induce cytotoxic effects on MG63 human osteosarcoma cells.

### 3.2. Effect of Rg1 on TA3-Induced Apoptosis in MG63 Human Osteosarcoma Cells

**Figure 2:**
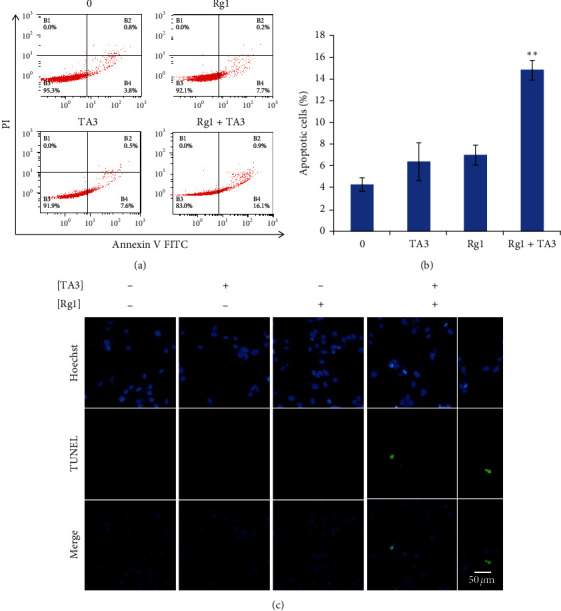
Effect of Rg1 on TA3-induced apoptosis in MG63 human osteosarcoma cells. (a) Apoptosis of MG63 cells was synergistically induced by Rg1 and TA3. TA3 (6 *μ*M) and/or Rg1 (250 *μ*M) were applied to the cells for 24 h. Then, Annexin V/PI apoptosis assay was performed. (b) The results from Annexin V/PI apoptosis assay were quantified. (c) DNA fragmentation was synergistically induced by Rg1 and TA3. TA3 (6 *μ*M) and/or Rg1 (250 *μ*M) were applied to the cells for 24 h. Then, the TUNEL assay was performed. The results were analyzed statistically by ANOVA and Tukey's test (^*∗∗*^*P*  <  0.01).

Based on the observations in the CCK-8 cell viability assay, I hypothesized the possible synergistic effect of Rg1 on TA3-induced apoptosis in MG63 cells. Therefore, I investigated the effect of Rg1 on TA3-induced apoptosis by the Annexin V/PI staining apoptosis assay. As seen in Figures 2(a) and 2(b), the total proportion of apoptotic cells (early cell death plus late cell death) significantly increased with the combined treatment of the two compounds. The synergistic effect of Rg1 on TA3-induced apoptosis of MG63 cells was 130%. This result indicates that Rg1 exerts a highly synergistic effect on TA3-induced apoptosis in MG63 cells.

To confirm DNA fragmentation in MG63 human osteosarcoma cells, terminal deoxynucleotidyl transferase- (TdT-) mediated dUTP nick-end labeling (TUNEL) assay was performed (Figure 2(c)). As visualized by green fluorescence (indicates DNA fragmentation) in fluorescence microscopy, Rg1 drove stimulation of TA3-induced DNA fragmentation.

### 3.3. Synergistic Effect of Rg1 and TA3 on Caspase-3 Activation

**Figure 3:**
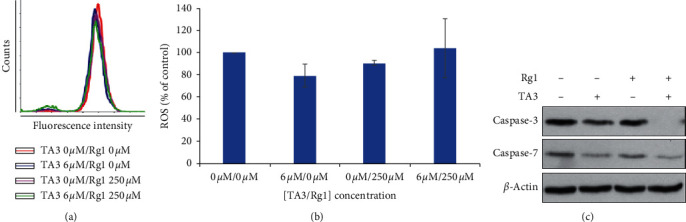
Synergistic effect of Rg1 and TA3 on caspase-3 activation. (a) Cellular ROS levels in MG63 cells incubated with TA3 (6 *μ*M) and/or Rg1 (250 *μ*M) were analyzed via the DCF-DA method. ROS levels were not changed significantly by the treatment of TA3 and/or Rg1. (b) Quantifications of DCF-DA ROS assay. (c) The level of the uncleaved form of caspase-3 decreased further when the cells were treated with both Rg1 (250 *μ*M) and TA3 (6 *μ*M) than with TA3 (6 *μ*M) alone while the uncleaved form of caspase-7 was not further affected by Rg1 (250 *μ*M) and TA3 (6 *μ*M) together than TA3 (6 *μ*M) alone.

An earlier study indicated that Rg1 reduces neuronal apoptosis by reducing the intracellular level of reactive oxygen species [18]. To investigate whether the synergistic effect of RG1 and TA3 is achieved by the ROS regulation, DCF-DA ROS assay was conducted. As shown in Figures 3(a) and 3(b), the cellular ROS levels were not affected by the treatments of Rg1, TA3, and Rg1 + TA3 (250 *μ*M, 6 *μ*M, and 250 *μ*M + 6 *μ*M, respectively) in a positive or negative way. This result may exclude the ROS regulation in the synergistic effect of Rg1 on TA3-induced apoptosis in MG63 human osteosarcoma cells. I also examined the effects of Rg1 and/or TA3 on caspase-3 and caspase-7, which are closely associated with the induction of cellular apoptosis, using western blot analysis. MG63 cells were treated with Rg1, TA3, and Rg1 + TA3 (250 *μ*M, 6 *μ*M, and 250 *μ*M + 6 *μ*M, respectively) for 24 h and western blot analysis was performed. Caspase-3 and caspase-7 can be activated by proteolysis to induce apoptosis [19]. As seen in Figure 3(c), the level of the uncleaved form of caspase-3 decreased further when the cells were treated with both Rg1 and TA3 than with TA3 alone while the uncleaved form of caspase-7 was not further affected by Rg1 and TA3 together than TA3 alone. This indicates that the synergistic apoptotic effect of the combination of Rg1 and TA3 is possibly achieved via selective caspase-3 regulation.

### 3.4. Synergistic Effects of Rg1 on TA3-Induced Antimetastatic Characteristics of MG63 Human Osteosarcoma Cells

**Figure 4:**
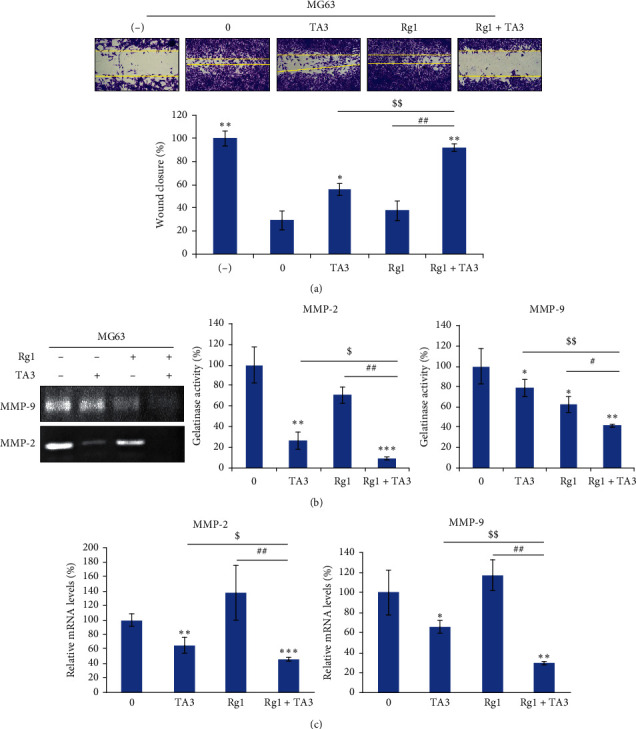
Synergistic effects of Rg1 on TA3-induced antimetastatic characteristics of MG63 human osteosarcoma cells. (a) Rg1 and TA3 synergistically attenuate migration of MG63 cells. Recovery of the wound width on the center of the cell surface was inhibited most when the cells were treated with Rg1 and TA3 together. Cells were treated with 0.1% FBS for negative control. The wound widths were measured for the quantitation. (b) Rg1 and TA3 synergistically inhibit gelatinase activities of both gelatinases (MMP-2 and MMP-9). (c) Transcriptional expressions of both enzymes (MMP-2 and MMP-9) were downregulated further by the combinatorial treatment of Rg1 (250 *μ*M) and TA3 (6 *μ*M) in MG63 cells. The results were analyzed statistically by ANOVA and Tukey's test (^*∗*^*P* < 0.05, ^*∗∗*^*P* < 0.01, ^*∗∗∗*^*P* < 0.001 vs control, ^$^*P* < 0.05 vs treatment of TA3, ^##^*P* < 0.01 vs treatment of Rg1).

Metastasis is the representative lethal process of cancer progression. For cancer cells to metastasize into the remote tissues of the human body, the acceleration of cancer cell migration is required [20]. To investigate the possible synergistic effects of Rg1 and TA3 on the migration of MG63 cells, wound healing assay was conducted. Cells were treated with Rg1, TA3, and Rg1 + TA3. As seen in Figure 4(a), the wound closure of Rg1 + TA3-treated cells was significantly inhibited compared with the cells treated with Rg1 or TA3 alone. This indicates that Rg1 and TA3, together, synergistically attenuate osteosarcoma cell migration.

Matrix metalloproteinases (MMPs) promote cancer metastasis by enzymatic degradation of the components of extracellular matrix (ECM) which blocks cells to move away. The major components include gelatin [21]. Out of various MMPs, MMP-2 and MMP-9 are the major gelatinases that can enzymatically degrade gelatin. The two enzymes are also able to contribute to the cancer cell migration nonproteolytically via their hemopexin domain [22]. TA3 was reported to inhibit those two MMPs via transcriptional regulation [14]. To identify whether Rg1 brings the synergy on this inhibition, gelatin zymography was performed. The areas of degraded gelatin became the narrowest when the samples were treated with Rg1 and TA3 together (Figure 4(b)). The result of quantitative real-time polymerase chain reaction (qRT-PCR) also indicates that the transcriptional expressions of the two enzymes were most severely downregulated when the cells were treated with both Rg1 and TA3 (Figure 4(c)). These data together implicate that the synergistic effect of RG1 on TA3-induced inhibition of the two gelatinases (MMP-2 and MMP-9) was achieved by transcriptional control of the two MMP genes. The results here together indicate that Rg1 stimulates TA3-induced antimetastatic effects in human osteosarcoma cells.

### 3.5. Effects of the Combination of Rg1 and TA3 on MAPKs and Transcription Factors in MG63 Human Osteosarcoma Cells

**Figure 5:**
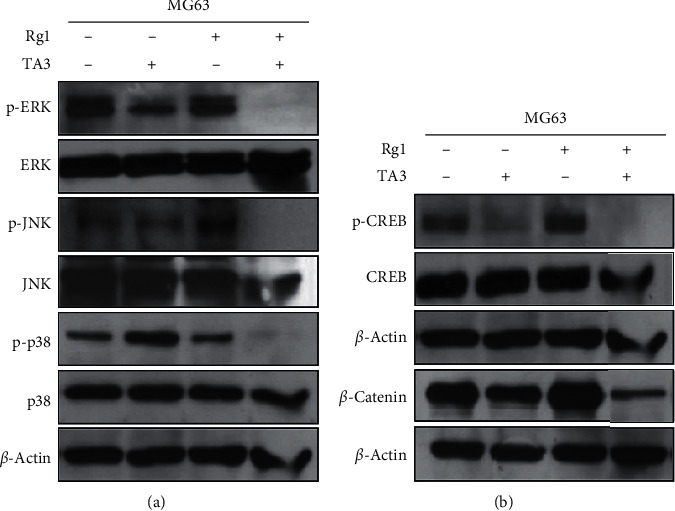
Effects of the combination of Rg1 and TA3 on signaling molecules in MG63 human osteosarcoma cells. (a) Activation of JNK, ERK, and p38, which are the representative MAPKs, is synergistically attenuated by Rg1 and TA3. (b) *β*-catenin and active form of CREB (p-CREB) were synergistically suppressed by Rg1 and TA3.

Mitogen-activated protein kinases (MAPKs) are the signaling molecules that are related to cell migration. They affect MMP expression, and JNK, ERK, and p38 are the three representative MAPKs [23]. Phosphorylated forms of those three proteins represent an active state. To test whether Rg1 exerts synergistic effects on the TA3-induced inhibitions of JNK, ERK, and p38, western blot analysis was performed with the MG63 cells treated with Rg1, TA3, and Rg1 + TA3 (Figure 5(a)).

The phosphorylated forms of JNK, ERK, and p38 were further decreased when Rg1 and TA3 were treated together than when TA3 was treated alone (Figure 5(a)). Two transcription factors, *β*-catenin and cAMP response element binding (CREB), which are known to be implicated with TA3 in MG63 cells, were also further suppressed when Rg1 and TA3 were treated together than when TA3 was solely treated as indicated by the decreased *β*-catenin and phosphorylated CREB (p-CREB) bands in western blot analysis (Figure 5(b)). This may indicate that Rg1 exerts its synergy on TA3 via those signaling pathways.

### 3.6. Synergistic Effects of Rg1 on TA3-Induced Cytotoxic Effect and Apoptosis in U2OS Human Osteosarcoma Cells

**Figure 6:**
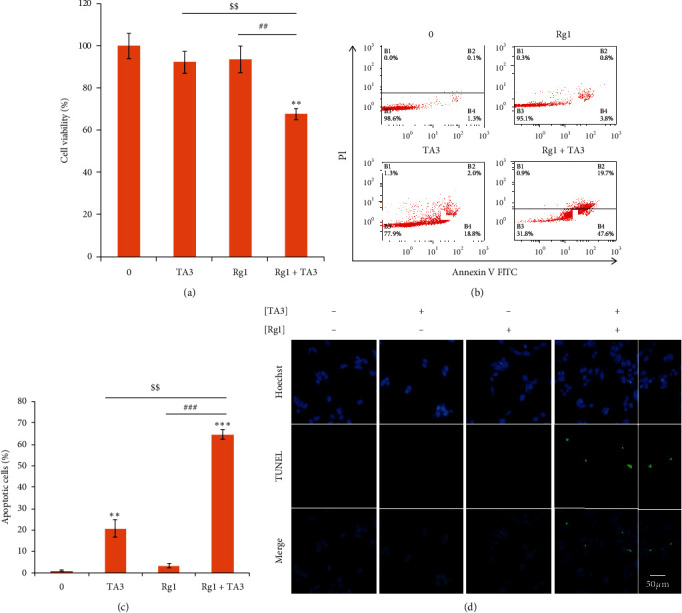
Stimulations of Rg1 on TA3-induced cytotoxic effect and apoptosis in U2OS human osteosarcoma cells. (a) Cytotoxic effect of Rg1 plus TA3 was monitored in U2OS cells. The cells were treated with each chemical compound for 24 h: TA3 (6 *μ*M), Rg1 (250 *μ*M), TA3 (6 *μ*M) plus Rg1 (250 *μ*M) for 24 h. Then, the samples were subjected to the cell viability assay using the CCK-8 kit. (b) Apoptosis of U2OS cells was synergistically induced by Rg1 and TA3. TA3 (6 *μ*M) and/or Rg1 (250 *μ*M) were applied to the cells for 24 h. Then, Annexin V/PI apoptosis assay was performed. (c) The results from Annexin V/PI apoptosis assay were quantified. (d) DNA fragmentation was synergistically induced by Rg1 and TA3. TA3 (6 *μ*M) and/or Rg1 (250 *μ*M) were applied to the cells for 24 h. Then, the TUNEL assay was performed. The results were statistically evaluated using ANOVA and Tukey's test (^*∗∗*^*P* < 0.01, ^*∗∗∗*^*P* < 0.001 vs control, ^$$^*P* < 0.01 vs treatment of TA3, ^###^*P* < 0.001 vs treatment of Rg1).

To validate whether the synergistic effects of Rg1 and TA3 are not specifically limited to MG63 cell line, the assays were conducted also in U2OS human osteosarcoma cells. Rg1 and TA3 exerted synergistic effects on cytotoxicity in U2OS cells (Figure 6(a)). The synergy was also in effect on the induction of apoptosis (Figures 6(b) and 6(c)). To confirm DNA fragmentation in U2OS human osteosarcoma cells, the TUNEL assay was also performed. As shown in Figure 6(d), Rg1 drove the stimulation of TA3-induced DNA fragmentation in U2OS cells. The results here indicate that the synergistic effects of Rg1 on TA3-induced cytotoxicity and apoptosis are not limited to the MG63 cells.

### 3.7. Synergistic Effects of Rg1 on TA3-Induced Antimetastatic Characteristics in U2OS Human Osteosarcoma Cells

**Figure 7:**
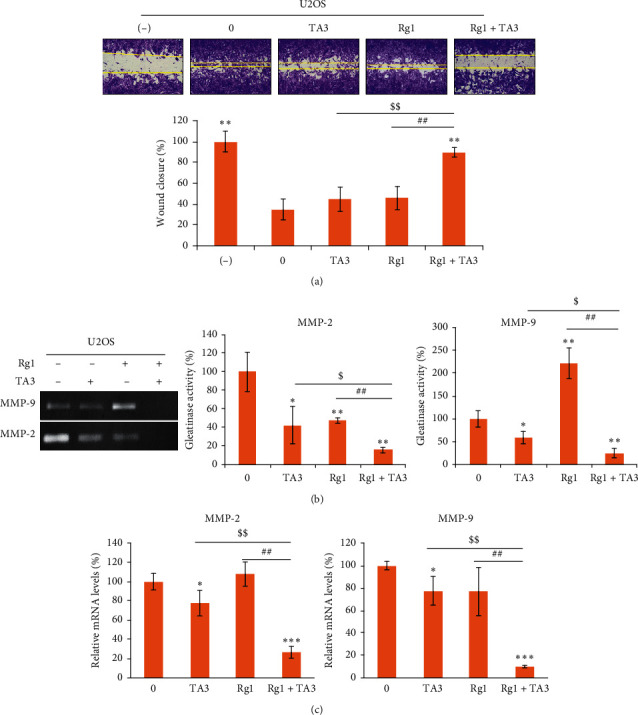
Stimulations of Rg1 on TA3-induced antimetastatic effects in U2OS human osteosarcoma cells. (a) Rg1 and TA3 synergistically attenuate the migration of U2OS cells. Recovery of the wound width on the center of the cell surface was inhibited most when the cells were treated with Rg1 and TA3 together. Cells were treated with 0.1% FBS for negative control. The wound widths were measured for the quantitation. (b) Rg1 and TA3 synergistically inhibit gelatinase activities of MMP-2 and MMP-9. (c) Transcriptional expressions of MMP-2 and MMP-9 were downregulated further by the combinatorial treatment of Rg1 (250 *μ*M) and TA3 (6 *μ*M) in U2OS cells. The results were analyzed statistically by ANOVA and Tukey's test (^*∗*^*P* < 0.05, ^*∗∗*^*P* < 0.01 vs control, ^$^*P* < 0.05 vs treatment of TA3, ^##^*P* < 0.01 vs treatment of Rg1).

Rg1 and TA3 also synergistically inhibited U2OS cell migration (Figure 7(a)). In addition, gelatinase activities of MMP-2 and MMP-9 were further attenuated when Rg1 and TA3 were treated together than when TA3 was treated alone (Figure 7(b)). The synergies on MMPs were achieved via transcriptional regulation (Figure 7(c)). These results together indicate that the synergistic effects of Rg1 and TA3 on antimetastatic effects are not limited specifically to the MG63 cell line.

## 4. Discussion

Ginseng has been widely used as a traditional medicine in northeast countries of Asia and ginsenosides are the major active compounds from ginseng. Among the various phytomedicinal effects of ginsenosides, anticancer effects have been rigorously investigated and reported so far [1]. Some examples are as follows: Rg3 and Rh2 both decrease viability and invasiveness of A549 human NSCLC cells while stimulating apoptosis significantly; Rb1 was reported to inhibit migration and invasion of liver cancer cells via Nrf2 regulation [1]. Among the various ginsenosides, Rg1 is a tetracyclic triterpenoid monomer. Rg1 has been known to affect various human organ systems including the immune, cardiovascular, and nervous systems with its pharmacological effects [7]. Notably, Rg1 reduces apoptosis of nerve cells and exerts protective effects on neurons [6]. In addition, a previous report indicated that Rg1 reduces the intracellular ROS level and protects neuronal cells from oxidative stress-induced apoptosis [18]. Here, in this study, I also investigated the effect of Rg1 on the ROS level in MG63 human osteosarcoma cells. The result indicated that Rg1 does not affect the cellular ROS level in MG63 cells. Also, Rg1 did not affect the apoptosis of human osteosarcoma cells when it was used alone. However, Rg1 dramatically stimulates apoptosis of human osteosarcoma cells when it is used in combination with TA3, an apoptosis-inducing compound in human osteosarcoma cells.

MDR is a major hurdle in cancer chemotherapy. Recently, ginsenosides have been reported to exert anti-MDR effects [1, 16]. A recent report indicated that a ginsenoside CK and maclurin, an active compound extracted from mulberry, exert a synergistic effect on the inhibition of MMP-1 in human keratinocyte cells [17]. Ginsenoside Rg3, together with gemcitabine, an antiangiogenic molecule which suppresses tumor growth, shows powerful inhibitory effects on angiogenesis and growth of lung cancer [24]. Rg3 and cisplatin also exert a synergistic effect on inhibition of the proliferation of cisplatin-resistant bladder tumor cells [25]. In addition, ginsenosides Rb1 and Rc showed a mild (around 50%) stimulatory effect on TA3-induced apoptosis in MG63 cells and this raised a possibility and a necessity of discovering an effective partner ginseng compound with strong synergy for antiosteosarcoma treatment [14]. On the basis of the observed synergistic effect of 130% on TA3-induced apoptosis, the combinatorial use of Rg1 and TA3 can be an effective complex agent for the development of a new antiosteosarcoma drug by overcoming MDR.

Many genes have been identified to be mutated to block apoptosis, cause cancer metastasis, and stimulate proliferation of tumors [26]. Among various cancer-related genes known so far, caspase-3 and caspase-7 were proposed to be related to TA3-induced apoptosis in MG63 cells [14]. These two caspases are initial signals that amplify the release of cytochrome c and trigger apoptosis [27, 28]. TA3 alone stimulates proteolytic cleavages of both caspase-3 and caspase-7 to convert the caspases to active status to promote the apoptosis of osteosarcoma cells. However, Rg1 alone does not stimulate any of the two caspases. When treated with TA3, Rg1 drives further stimulation of the activation of only caspase-3, selectively, while it does not show an additive effect on caspase-7 activation through the proteolytic cleavage. This suggests that Rg1 may exert the synergy on TA3-induced apoptosis via caspase-3 regulation.

**Figure 8:**
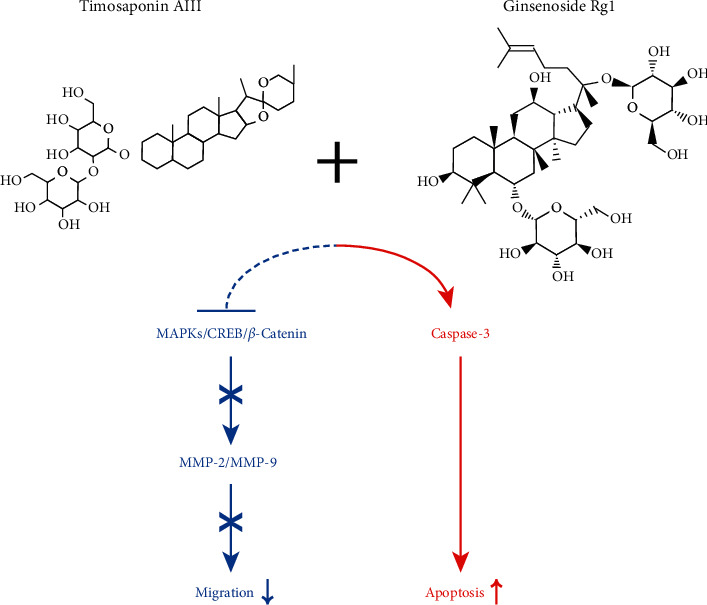
Summary of synergistic antiapoptotic and antimetastatic effects exerted by Rg1 and TA3 via modulation of MAPK, CREB, *β*-catenin, and caspase-3 signaling pathways in MG63 human osteosarcoma cells. Rg1 intensified TA3-induced activation of caspase-3. This regulation would lead to the stimulation of apoptosis induction. A combination of Rg1 and TA3 inhibited MAPKs (JNK, ERK, and p38), CREB, and *β*-catenin signaling pathways. This regulation would result in the inhibition of MMP-2 and MMP-9 and suppression of cell migration in human osteosarcoma cells.

Cancer metastasizes through multiple processes. Out of those processes, cancer cell migration and ECM degradation are key steps to get into the blood and lymphatic vessels to reach the various types of tissues in distance. A previous study reported that TA3 attenuates the migration of MG63 osteosarcoma cells via the inhibitions of gelatinases (MMP-2 and MMP-9) via transcriptional suppression [14]. Rg1 also shows synergistic effects on the TA3-induced inhibitions of the three representative MAPKs (JNK, p38, and ERK) and the transcription factors (*β*-catenin and CREB), which are key regulators of MMPs. This may indicate that the synergy between Rg1 and TA3 is effective in a broad range of cancer characteristics beyond cytotoxicity and apoptosis. The synergy is exerted via negative regulation of MAPK, CREB and *β*-catenin, and positive regulation of caspase-3 (Figure 8). Considering that the synergies are not limited to MG63 cells and also observed in U2OS human osteosarcoma cells, the development of the antiosteosarcoma agent based on the complex application of Rg1 and TA3 may be an effective approach to the new drug for human osteosarcoma.

## 5. Conclusion

In this study, I found that ginsenoside Rg1 generates a synergistic effect on apoptosis, which was induced by TA3 in MG63 and U2OS human osteosarcoma cells. The synergies are also effective in antimetastatic virtues in both human osteosarcoma cells. For their broad range of synergistic effect on various characteristics of osteosarcoma, the results presented in this study may open the opportunity for ginsenoside Rg1 to be used as a partner molecule of TA3 for the development of new antiosteosarcoma agents and thus may have practical value for utilization in the pharmaceutical and the food industry sectors.

## Figures and Tables

**Table 1:** List of primer sequences used for qRT-PCR.

Gene	Sequence
MMP-2	Forward primer: 5′-TTGACGGTAAGGACGGACTC-3′ Reverse primer: 5′-ACTTGCAGTACTCCCCATCG-3′

MMP-9	Forward primer: 5′-GAGACCGGTGAGCTGGAT-3′ Reverse primer: 5′-TACACGCGAGTGAAGGTGAG-3′

GAPDH	Forward primer: 5′-TGCACCACCAACTGCTTAGC-3′ Reverse primer: 5′-GGCATGGACTGTGGTCATGAG-3′

## Data Availability

The data used to support the conclusions of this study are available upon request by contacting the corresponding author.
